# Cytotoxic potentials of silibinin assisted silver nanoparticles on human colorectal HT-29 cancer cells

**DOI:** 10.6026/97320630016817

**Published:** 2020-11-30

**Authors:** Kiren Jackson, Ezhilarasan Devaraj, Thangavelu Lakshmi, Shanmugam Rajeshkumar, Kamal Dua, Dinesh Kumar Chellappan, Subramanian Raghunandhakumar

**Affiliations:** 1 Department of Pharmacology, Saveetha Dental College, Saveetha Institute of Medical and Technical Sciences (SIMATS), Chennai, Tamil Nadu, India 600 077; 2 Discipline of Pharmacy, Graduate School of Health, University of Technology Sydney, Sydney, NSW 2007, Australia; 3 School of Biomedical Sciences and Pharmacy, University of Newcastle, Newcastle, NSW 2308, Australia; 4 Department of Life Sciences, School of Pharmacy, International Medical University, Bukit Jalil 57000, Kuala Lumpur, Malaysia

**Keywords:** colorectal cancer, silibinin, silver nanoparticles, apoptosis

## Abstract

It is of interest to study the cytotoxicity of silibinin assisted silver nanoparticles in human colorectal (HT-29) cancer cells. Silver nanoparticles were synthesized using silibinin as a reducing agent. The synthesized silibinin assisted silver nanoparticles ( SSNPs) were characterized and analyzed using a transmission electron microscope and spectrophotometer. The SSNPs synthesized in this study are spherical and their size ranges from 10 to 80 nm. HT-29 cells were treated with different concentrations (2, 4, 6, 8 and 10 ng/mL) of SSNPs and cytotoxicity was evaluated. The apoptosis was using flow cytometry. p53 protein expression using western blot. SSNPs are induced a decrease in viability and increased concentration-dependent cytotoxicity in HT-29 cells. SSNPs treatment also caused apoptosis-related morphological changes. SSNPs treatments at 8 and 16 ng/ml showed a prominent apoptotic change i.e., 70.3% and 83.6% respectively, and decreased viability of HT-29 cells 20% and 11.2% respectively as compared to control cells. SSNPs treatments induced p53 expression in HT-29 cells. Data shows that SSNPs have the potential to induce apoptosis in colorectal cancer cells. This provides insights for the further evaluation of SSNPs in fighting colon cancer.

## Background

Colorectal cancer (CRC) kills almost 700,000 people annually, making it one of the second leading cause of cancer-related deaths worldwide and it also accounts for approximately 10% of cancer-related mortality in Western countries [1- [3]. Various factors have associated with progression of colon cancer including mutation, bacterial infection and irradiation [4-6]. The colon cancer incidence is increasing in several countries due to the pervasive adoption of the Western diet and lifestyle [7]. For instance, high and frequent intake of processed and red meats, preserved foods, saturated/animal fats, cholesterol, high sugar and spicy foods, tubers, or refined carbohydrates have been positively associated with CRC risk [8]. Treatment modalities for primary and metastatic CRC are laparoscopic surgery for primary disease, radiotherapy for rectal cancer, and neoadjuvant and palliative chemotherapies [9]. Targeted therapies with anti-epidermal growth factor receptor have also been tried for CRC [10]. Despite significant clinical management involving targeted therapies, chemo/radiotherapies, and surgical procedures, CRC remains one of the frequent causes for cancer-related death worldwide and it is cause for concern [11]. Further, chemo/radiotherapy is responsible for off-target side effects like nausea, vomiting, diarrhea, mucositis with taste alteration, alopecia, constipation, fatigue, anorexia, sleep disturbance, headache, anemia and dry skin [12- 13]. Therefore, the need of the hour is to identify a therapeutic compound for use in CRC patients with fewer or no side effects. Nanoparticles based phytomedicines are increasingly used as anticancer medicines [14- 15]. Studies showed that various types of metallic and metal oxide nanoparticles are employed for the analysis of anticancer potential against HT-29 and other gastrointestinal cancer cell lines [16- 18]. Various metal oxide NPs, including silver oxide, cobalt oxide, manganese oxide, titanium dioxide, and zinc oxide, have been investigated for their anticancer activities [19-21].

Silibinin is a flavonolignan isolated from the fruit and seeds of the medicinal plant Silybum marianum commonly called Milk thistle [22] and it is one of the most effective flavonoid compound tested against a variety of hepatic disease [22]. Silibinin was also shown to have anticancer, antimicrobial, antioxidant, and membrane-stabilizing properties [13,22-30]. Silibinin belongs to the group of Biopharmaceutics Classification System class II drug and has been proven to be very potential drug candidate [31]. However, the clinical applications of silibinin show some limitations due to its low aqueous solubility and bioavailability, poor penetration into the epithelial cells of the intestine, high metabolism, and rapid systemic elimination [22,32]. Nonetheless, a nanotechnology-based drug delivery system explores the great potential for phytochemicals to enhance their aqueous solubility, improve stability, and the pharmacological activity [32]. In recent years, silver nanoparticles have attracted considerable interest worldwide to develop a new-generation treatment tool for cancer because of their ability to kill cancer cells [17,33,34]. Therefore, it is of interest to study the cytotoxicity of silibinin assisted silver nanoparticles in human colorectal (HT-29) cancer cells.

## Methodology:

### Chemicals:

Dulbecco's minimum essential low glucose medium (DMEM), dimethylsulfoxide (DMSO), penicillin, streptomycin, trypsin-ethylenediaminetetraacetic acid (EDTA), 3-(4,5-dimethylthiazol-2-yl)-2,5-diphenyltetrazolium bromide (MTT), fetal bovine serum (FBS) were obtained from GIBCO BRL (Gaithersburg, MD). Silibinin and silver nitrate were purchased from M/s. Sigma chemical (Chennai, India). All other chemicals purchased locally and were of analytical grade.

### Cell culture and maintenance:

The HT-29 cell line was procured from National Centre for Cell Science (Pune, Maharashtra, India). The cells were cultured in 25 cm2 flask using DMEM with low glucose containing 10% FBS with penicillin (100 units/mL) and streptomycin (100 µg/mL) in a standard humidified atmosphere with 5% CO2 at 37°C. After acclimatization for a couple of passages, cells were used for experiments. Once the cells were reached enough confluence, they were detached using the trypsin-EDTA solution (0.25%) and were seeded for experiments. All the experiments were performed with 70-80% confluence.

### Nanoparticle preparation and characterization:

Hundred mg of silibinin mixed with 25 mL of double distilled water and boiled for 2 min and then 1 mM of silver nitrate (80 mL) was added to 20 mL silibinin solution and kept in magnetic stirrer for nanoparticles synthesis. The color change was observed visually and absorbance was taken from 300-600 nm periodically by UV-visible spectrophotometry. The synthesized nanoparticles centrifuged and dried for the morphology analysis using a transmission electron microscope. The sizes of the nanoparticles were measured using ImageJ software. The SSNPs were dissolved in 0.1% DMSO and used in the following experiments.

### MTT assay:

MTT assay was performed to assess the SSNPs-induced cytotoxicity in HT-29 cells [35]. Briefly, cells were seeded at 1 x 104 cells/well in 96-well plates. 24 h later, the existing medium was changed with medium containing different concentrations of SSNPs (2, 4, 6, 8, and 10 ng/mL) and incubated for 24 h. After 24 h, the media was aspirated and cells were washed with phosphate buffered saline (PBS) once and then cells were incubated with 50 µL of MTT (0.5 mg/mL) for 4 h inside the CO2 incubator. After incubation, MTT was discarded and DMSO was added to wells to dissolve the colored formazan crystals produced by the viable cells. The purple-blue formazan formed was measured using Perkin Elmer Multimode Reader (USA) at 570 nm. The optical density of each sample was compared with control optical density.

### Calcein-AM staining assay:

Cells were treated with 8 and 16 ng/mL of SSNPs and cell viability was analyzed by calcein-AM for live cell staining according to the manufacturer's instruction (BD Biosciences, San Jose, CA). Calcein-AM is a highly lipophilic membrane-permeable vital dye that readily enters viable cells and is converted to calcein by esterase present in the intracellular domain, which produces an intense green fluorescent. Thus, the green color intensity indicates the viability of cells. Briefly, after treatments cells were washed twice with PBS and were stained with calcein-AM (0.2 µM) and incubated for 30 min at 37°C and then images were captured using Nikon Eclipse Ti fluorescence microscope (Nikon Instruments Inc., NY, USA).

### Morphological analysis of apoptosis by annexin V staining:

HT-29 cells were seeded (1 x 104 cells/well) in 96 well plates and after the cell adherence for 24 h; cells were treated with different concentrations of SSNPs and incubated for 24 h. At the end of the treatment period, cells were collected and fixed in 4% paraformaldehyde and subsequently stained with FITC (fluorescein isothiocyanate)-annexin V kit (BD Biosciences, San Jose, CA) for 15 min. Then cells were washed thrice with PBS and representative fields were captured immediately using Nikon Eclipse Ti fluorescence microscope (Nikon Instruments Inc., NY, USA).

### Apoptosis analysis by flow cytometry:

Briefly, 1 x 106 cells were seeded in 6 well plates and incubated for adherence. Then cells were treated with different concentrations of SSNPs for 24 h. The apoptotic cells were quantified by annexin V-FITC/propidium iodide (PI) co-staining assay. Briefly, at the end of the 24 h incubation, the cells were harvested and centrifuged at 1800 rpm for 8 min. The pellet was suspended in 50 µL of binding buffer containing 0.5 µL of annexin V-FITC and then incubated at 4°C for 30 min in the dark. PI (50 µg/mL) in 200 µL binding buffer was added and incubated for 5 min. The cells were analyzed in the flow cytometer (CyAn ADP Analyzer, Beckman Coulter, USA) [36].

### Western blot analysis: 

After treatments cells were collected and lysed using RIPA buffer containing phosphatase inhibitor cocktails. Total protein estimation was done using bovine serum albumin (BSA) as standard. Electrophoresis was done with protein extracts and electroblotted onto polyvinylidene difluoride membrane. Then the membrane was blocked with 5% BSA and incubated overnight with p53 (monoclonal, IgG1) primary antibody at 4°C and 2 h with the corresponding secondary antibodies at room temperature. The enhanced chemiluminescence with protein A-horseradish peroxidase was used to detect the immunoreactive bands.

### Statistical analysis:

Data were expressed as mean ± S.E.M and analyzed by one-way ANOVA followed by Dunnett's multiple comparison tests to determine the significant differences between groups. p<0.05 was considered as significant (Graph Pad prism 7.0. CA, USA).

## Results:

**Figure 1:**
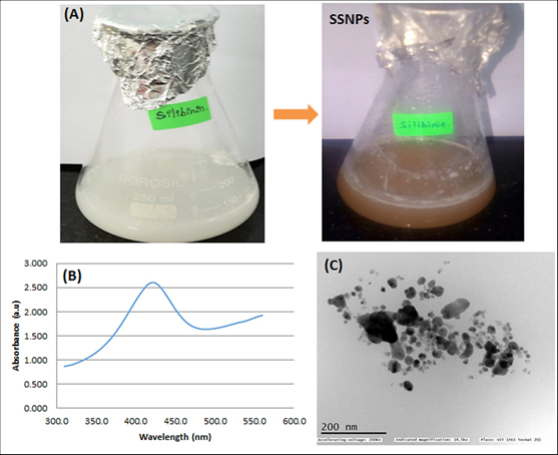
Characterization of silibinin assisted silver nanoparticles (SSNPs).(A) Visual transmission of SSNPs; (B) UV-Vis spectra of biosynthesized SSNPs; (C) Transmission electron microscope analysis of SSNPs.

**Figure 2:**
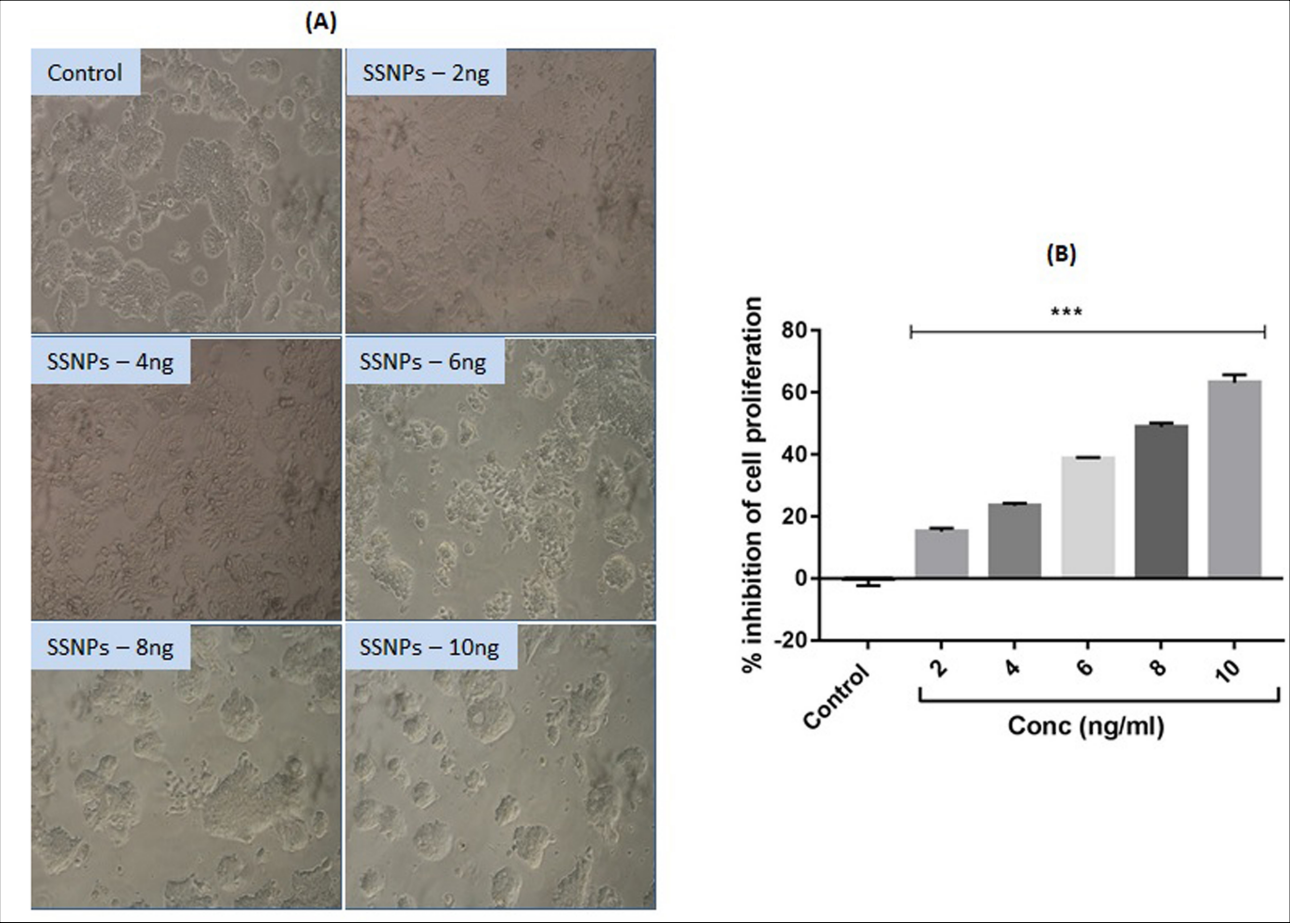
Silibinin mediated silver nanoparticles (SSNPs) induced changes in the proliferation of HT-29 cells. A. Morphology of control and SSNPs treated HT-29 cells (scale bar: 100 µm). B. Cytotoxicity analysis by MTT assay. n=3, ***p< 0.001 vs control.

In this study, we have used silibinin as a reducing agent to synthesize SSNPs. The visual color changes to brown color confirmed the syntheses of SSNPs (Figure 1A). The optical properties of spherical SSNPs are highly dependent on the nanoparticle diameter. In this study, the bioreduction of the silver ions was monitored spectro-photometrically at 420 nm. Our UV-visible spectra showed smaller, spherical SSNPs, which primarily absorbed light and had peaks near 420 nm (Figure 1B). The transmission electron microscope analysis confirmed that the synthesized nanoparticles are mostly spherical and their sizes were ranges from 10 to 80 nm (Figure 1C). The morphology of SSNPs treated HT-29 cells are presented in Figure 2A. HT-29 cells are observed with typical clusters of colonies. Treatment with increasing concentrations (2, 4, 6, 8 and 10 ng/mL) of SSNPs significantly (p<0.001) inhibited the proliferation of HT-29 cells in a dose-dependent manner (Figure 2B). The IC50 value of SSNPs for HT-29 cells was found to be 8 ng/mL. Therefore, further studies were carried out with the concentration ranges of 8 and 16 ng/mL.

**Figure 3:**
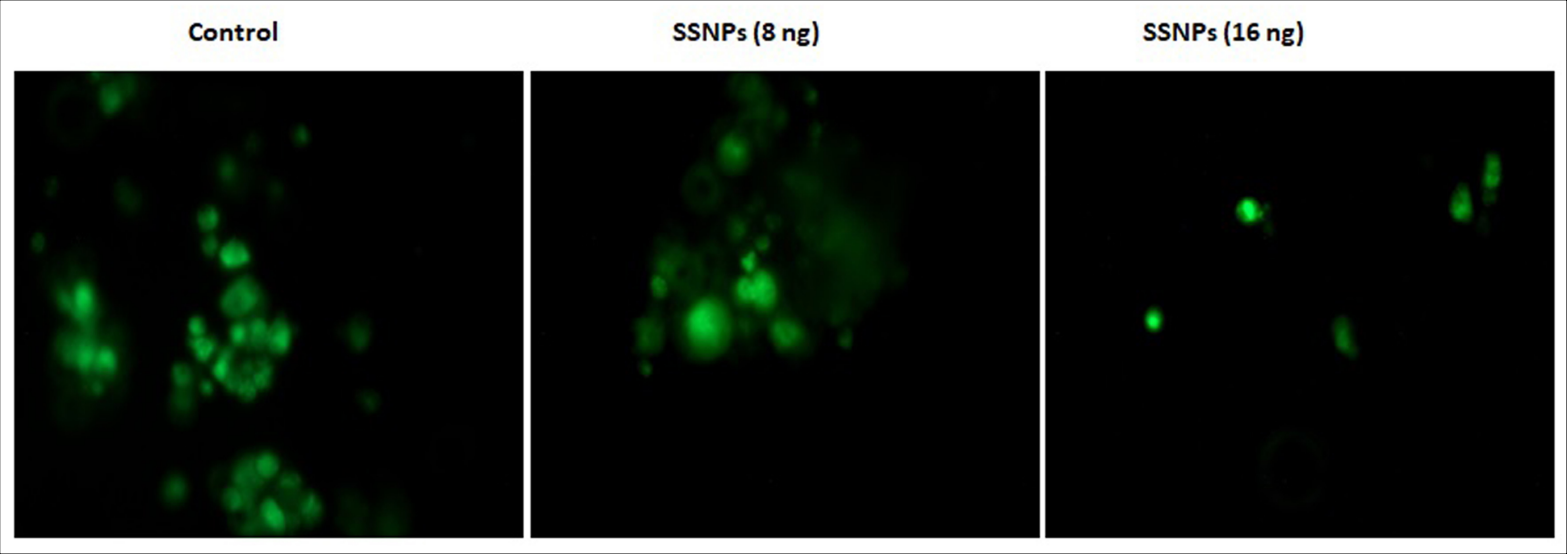
Representative fluorescence microscope images (scale bar: 100 µm) show cell viability assessed by calcein AM staining in control and silibinin mediated silver nanoparticles (SSNPs) treated HT-29 cells.

**Figure 4:**
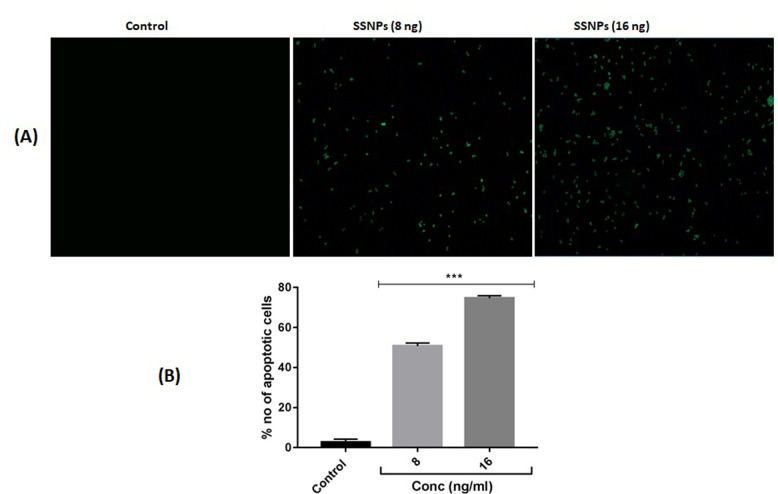
(A) Morphological analysis of apoptosis by annexin v staining (4X) (scale bar: 100 µm).(B). Quantification of apoptotic cells. ***p<0.001 vs control.

**Figure 5:**
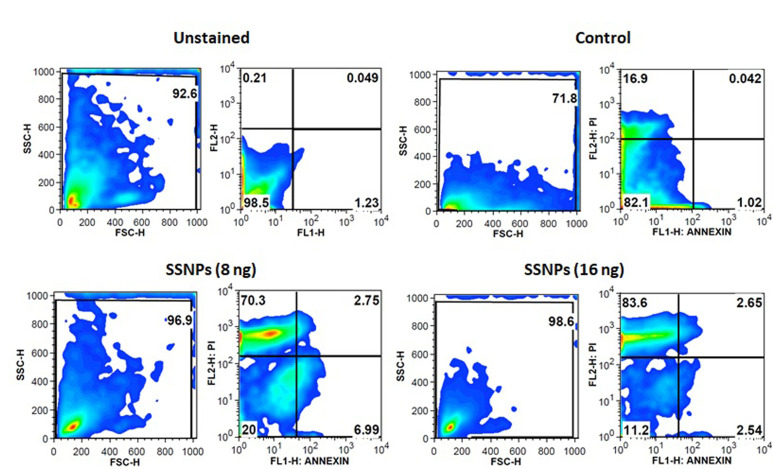
Flow cytometric analysis of apoptosis using annexin v and propidium iodide staining. Top left quartile percentage indicates the presence of apoptotic cells.

**Figure 6:**
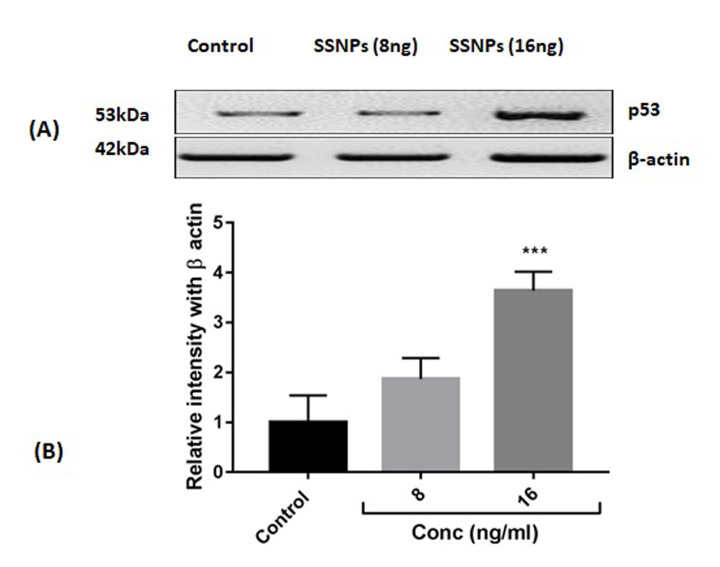
(A) Western blot expression of p53. (B). Quantification of p53 protein expression by densitometry analysis. n=3.***p<0.001 vs control.

Furthermore, the cytotoxic effect of SSNPs on HT-29 cells was confirmed by calcein-AM staining. HT-29 cells were treated with two different concentrations (8 and 16 ng/mL) of SSNPs for 24 h and live and dead cells were labeled using fluorescent probes calcein AM and photographs were taken under the fluorescence microscope. The results demonstrated that the SSNPs are decreasing green fluorescence in a dose-dependent manner, which indicates decreased viability of HT-29 cells (Figure 3). Annexin v staining is one of the gold standard methods to analyze the apoptotic changes morphologically. SSNPs treatments in HT-29 cells caused a dose-dependent increase in annexin v positive cells (Figure 4A). The percentage of annexin v positive cells was increased in a concentration-dependent manner as compared to control cells (p< 0.001) (Figure 4B). The apoptosis-inducing potential of SSNPs also analyzed through flow cytometry by annexin v and propidium iodide staining. The unstained (without annexin v and PI) control cells (with annexin v and PI without SSNPs treatments) did not show any significant change in their viability and they were 98.5% and 82.1% viable respectively. SSNPs treated cells at 8 and 16 ng/ml showed a prominent apoptotic change i.e., 70.3% and 83.6% respectively, and decreased viability to 20% and 11.2% respectively (Figure 5). The tumor suppressor protein p53 expression was analyzed in cells treated with SSNPs. SSNPs treatments only in high dose caused a significant increase in the p53 protein expression when compared to control cells (Figure 6A). The quantification of p53 expression also confirmed the significant increase (16 ng/mL vs p<0.001) upon SSNPs treatments (Figure 6B).

## Discussion:

Silver nanoparticles range from 1 and 100 nm in size has unique properties, which help in molecular diagnostics and therapies [33]. Silver nanoparticles are mainly synthesized by the physical and chemical methods. The main drawback of the chemical and physical methods of silver nanoparticle formation is that they are extremely costly and also involve the use of toxic, hazardous chemicals and they contain potential environmental and biological risks [33, 37]. To overcome this, the biological method provides a feasible alternative [38]. In our recent study, we have showed β-Sitosterol, a phytocompound can be used as a good reducing agent for synthesis of silver nanoparticles [17]. Phytomediated silver nanoparticles were synthesized using Silybum marianum seed extract [39]. In this study, we have synthesized spherical silver nanoparticles using a phytocompound compound silibinin, and tested their cytotoxic potential in HT-29 cells. In previous studies, silibinin was shown to have apoptosis and cell cycle arrest potential in HT-29 cells [40, 41]. Similarly, silver nanoparticles synthesized from biological and chemical methods have also shown to have apoptosis-inducing potential in HT-29 cells [42,43]. In this study, silibinin-mediated silver nanoparticles induced dose-dependent cytotoxicity in HT-29 cells. In previous studies, silibinin treatments caused cytotoxicity at 50–100 µg/ml [40,41] and however, in this study, as compared to silibinin, SSNPs showed prominent cytotoxic effect at nanogram levels (8-16 ng/ml) in HT-29 cells. The results of the current study suggest that SSNPs are effective against colon cancer cells even at nanogram levels. Cell viability can be measured using the fluorescent probe calcein-AM, which can differentiate between living and dead cells [44]. In viable HT-29 cells, intracellular esterase can convert calcein-AM to calcein, which stays in living cells and emits intense green fluorescence [45]. In view of these reports, the current result suggests that decreased green fluorescence intensity in SSNPs treated HT-29 cells is due to fall in cell viability, which further supports the toxicity induced by SSNPs.

Apoptosis is one of the commonly reported mechanisms for silver nanoparticles induced cytotoxicity in human breast, lung, liver, skin and oral cancer cell lines [46-50]. Membrane damage is often reported when the cancer cells are treated with silver nanoparticles [49,51] and therefore, morphological changes related to apoptosis were investigated by annexin v staining. In the early apoptotic stage, phosphatidylserine residues expose from the inner to the outer leaflet of the plasma membrane and annexin V precisely bind to the exposed phosphatidylserine residues and therefore, it was used as a specific probe for morphological investigation of apoptosis [35,52]. In our present result presence of green color fluorescence in the experimental group indicating the membrane damaged cells undergoing apoptosis. The flow cytometry analysis also confirms the reduction of viability after SSNPs treatments, which further support the apoptosis-inducing potentials of SSNPs in HT-29 cells.

Apoptosis is the most noticeable biological outcome of p53 activation in cell culture experiments as suggested by Chen (2016) [53]. p53 is a transcription factor and it can be activated by several stress signals including DNA damage, apoptosis, nutrient deprivation, and cell-cycle arrest [13,27,54]. p53 has the ability to induce apoptosis by transcription-dependent and -independent manner [55]. Transcriptionally p53 activates the pro-apoptotic Bcl-2 family proteins and suppresses the anti-apoptotic Bcl-2 family proteins. p53 directly interacts with Bax that successively stimulates the release of cytochrome C via mitochondrial outer membrane permeabilization and aid in the induction of apoptosis through caspase activation [53,56,57]. It is likely that the apoptosis inducing potentials of SSNPs observed in this study could be due to the transcriptional activation of p53 protein expression in HT-29 cells.

## Conclusion

Silibinin assisted silver nano-particles had a spherical shape with an average size of 10 to 80 nm. SSNPs treatments induced dose-dependent cytotoxicity with a concomitant decrease in the cell viability in human colorectal cancer cell line. SSNPs treatment induced p53 protein expression and this could be the possible reason behind the apoptotic changes observed in this study. Thus, SSNPs is a potential therapeutic candidate for in vivo evaluation in the fight against colon cancer.
